# Two-year outcome data suggest that less invasive surfactant administration (LISA) is safe. Results from the follow-up of the randomized controlled AMV (avoid mechanical ventilation) study

**DOI:** 10.1007/s00431-020-03572-0

**Published:** 2020-02-18

**Authors:** Egbert Herting, Angela Kribs, Christoph Härtel, Axel von der Wense, Ursula Weller, Thomas Hoehn, Matthias Vochem, Jens Möller, Christian Wieg, Bernhard Roth, Wolfgang Göpel, for the German Neonatal Network (GNN)

**Affiliations:** 1 Department of Paediatrics, University Hospital of Schleswig-Holstein, University of Lübeck, Ratzeburger Allee 160, D-23538 Lübeck, Germany; 2 Department of Neonatology, University of Cologne, Cologne, Germany; 3 Department of Neonatology, Children’s Hospital Hamburg-Altona, Hamburg, Germany; 4 Department of Paediatrics, Evangelical Klinikum Bethel, Bielefeld, Germany; 5 Department of Paediatrics, University of Düsseldorf, Düsseldorf, Germany; 6 Department of Neonatology, Olgahospital Stuttgart, Stuttgart, Germany; 7 Department of Paediatrics, Saarbrücken General Hospital, Saarbrücken, Germany; 8 Germany Children’s Hospital Aschaffenburg-Alzenau, Aschaffenburg, Germany

**Keywords:** Less invasive surfactant, LISA – CPAP, Premature infants, Outcome

## Abstract

Less invasive surfactant administration (LISA) is a method to deliver surfactant to spontaneously breathing premature infants via a thin catheter. Here we report the two-year outcome from the AMV (avoid mechanical ventilation) study, the first randomized controlled trial on this mode of surfactant delivery. No statistically significant differences in weight, length or neurodevelopmental outcome (Bayley II scores) were found between the LISA intervention group (*n* = 95) and the control group (*n* = 84) that received standard treatment.

*Conclusion*: No differences in outcome were observed at 2 years. LISA seems safe in that aspect.

**Table:** 

**What is Known:** • *LISA is a method that is in increasing use for surfactant delivery to spontaneously breathing infants. LISA reduces the need for mechanical ventilation.*	
**What is New:** • *Outcome data at 2 years from the first randomized study with LISA raise no safety concerns in comparison to a group of infants that received standard treatment.*

**What is Known:**

• *LISA is a method that is in increasing use for surfactant delivery to spontaneously breathing infants. LISA reduces the need for mechanical ventilation.*

**What is New:**

• *Outcome data at 2 years from the first randomized study with LISA raise no safety concerns in comparison to a group of infants that received standard treatment.*

## Introduction

Less invasive surfactant administration (LISA) allows to deliver surfactant to infants under spontaneous breathing with CPAP (CPAP = Continuous positive airway pressure) support without the use of positive-pressure ventilation (1). The method meets increasing clinical interest (2–4) and is recommended now both in national (5) and international guidelines for surfactant replacement therapy (6). Initially, there were concerns about safety as LISA needs manipulations including laryngoscopy and introduction of a thin catheter into the trachea at a vulnerable time point soon after birth.

However, recent meta-analyses (7) indicate that LISA reduces the need for mechanical ventilation and probably also decreases the incidence of intraventricular haemorrhage (IVH) and bronchopulmonary dysplasia (BPD). In this meta-analysis, LISA was shown to be more effective than CPAP alone or short-term endotracheal intubation/INSURE (INSURE = Intubate surfactant extubate) for surfactant delivery (7). However, follow-up data on infants that have received LISA treatment are sparse.

The aim of the current study was to report the 2-year follow-up from the first randomized controlled multicentre study (8) on LISA, the AMV study (AMV = Avoid mechanical ventilation).

## Methods

In the AMV study, infants with a gestational age between 26 and 28 weeks were included and received surfactant by LISA when the oxygen demand (FiO_2_) exceeded 30%. Controls received standard therapy with the option of endotracheal intubation intratracheal bolus rescue surfactant under mechanical ventilation. The primary endpoint, a reduction in the need for mechanical ventilation at 72 h of life, was reached (22 vs. 46%, *p* = 0.008). LISA also significantly reduced the need for mechanical ventilation during the whole hospital stay (33 vs. 73%, *p* < 0.0001) as well as the duration of oxygen therapy in comparison to standard treatment. There was a reduction in the need for oxygen at day 28 (30 vs. 45%) in favour of the LISA group and a trend towards a reduction in the rate of BPD at 36 weeks (8 vs. 14%, *p* = 0.27), but these were not the primary endpoints. Other complications (e.g. IVH grade III/IV: 7 vs. 5%, *p* = 0.59) and serious adverse events (SAE) overall were not different between the groups (19 vs. 25%, *p* = 0.34). However, the AMV study was not powered to demonstrate a reduction in chronic lung disease or other relevant complications of premature birth (8).

The initial design of the AMV study did not include the planning of a follow-up study. However, whilst the AMV study was in progress, the German government (GBA = Gemeinsamer Bundesausschuss) mandated a 2-year follow-up with Bayley II scales for all infants below 1500 g. In addition, at that time, the German Neonatal Network (GNN) was founded with the aim to study short- and long-term complications and the outcome of very low birth weight infants (VLBW = very low birth weight infants) in the time span until 2021. This gave us the possibility to follow up the AMV study participants at 2 years.

Towards the end of the AMV study, we contacted all actively contributing centres whether they would be willing to collect and deliver data from the mandatory follow-up at 2-year corrected age. An addendum to the ethical approval for the additional data collection was sought, and the parents were contacted and asked for additional information on the course after discharge from the hospital (e.g. about stays in hospital and complications like bronchitis, visual and hearing impairment). A protocol/CRF for the follow-up asking for somatic data and Bayley scales of infant development (Bayley II scores), classified into mental development index (MDI) and the psychomotor development index (PDI), was filled in by the respective centres.

For the statistical analysis of the data, t-test and Fisher’s exact test were used.

## Results

**Figure 1:**
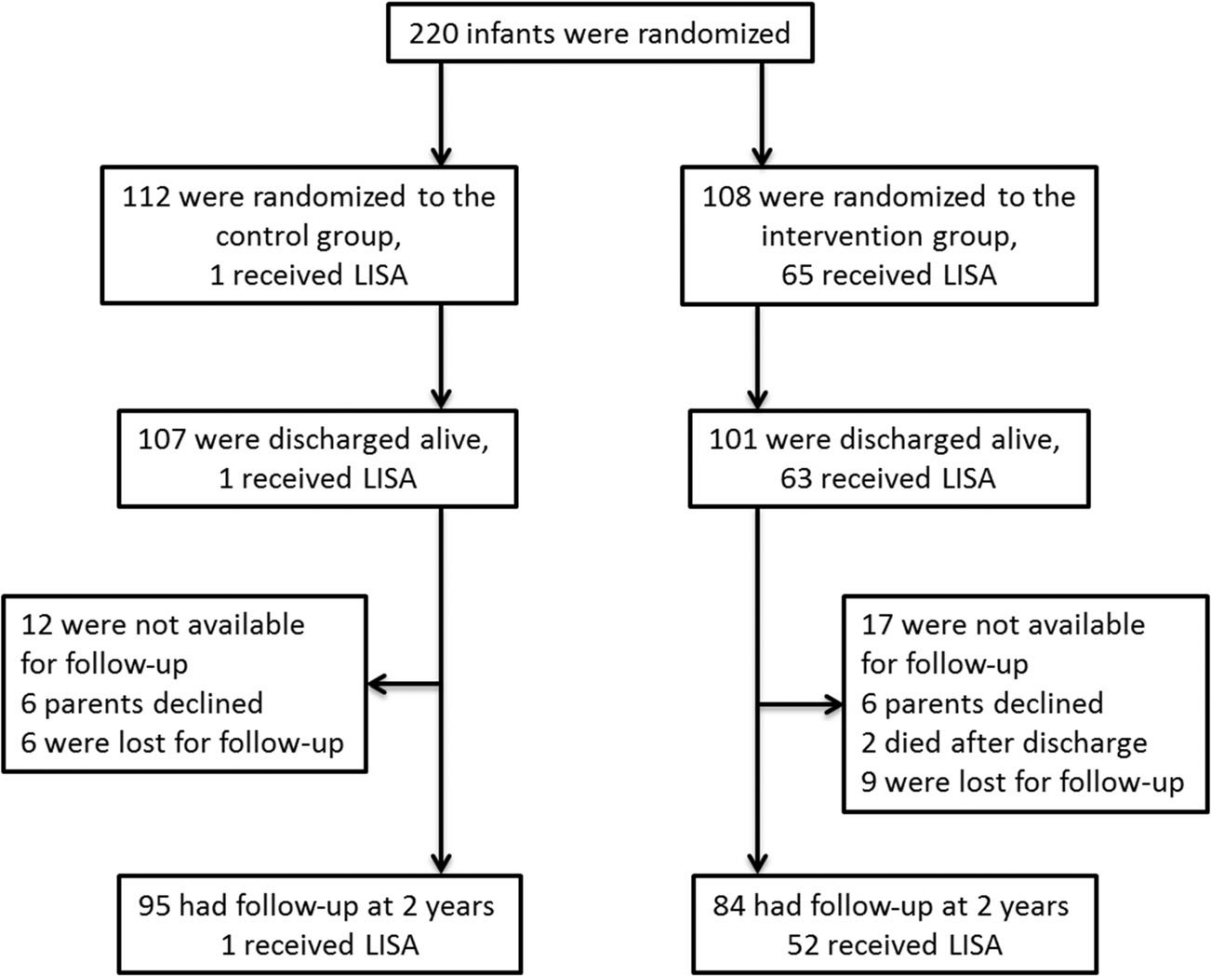
Patient numbers included in the follow-up. 1 patient in the control group received Less Invasive Surfactant Administration (LISA), but was kept in the study (intention to treat analysis).

In the AMV study, 112 infants were originally randomized to the control and 108 infants to the LISA intervention group (8). One hundred seven infants in the control group survived until discharge, and 95 infants were available for the follow-up. In the intervention group, 80 infants received surfactant, 65 by the LISA method, 15 received surfactant via an endotracheal tube (e.g. following intubation in the delivery room due to low APGAR or severe respiratory distress), and 28 infants received no surfactant as their FiO_2_ did not exceed 30%. In the intervention group, 101 survived to discharge, and 84 infants were followed-up (see Fig. 1).

At 2-year corrected age (27.1 ± 2.4 vs. 27.5 ± 3.4 months), growth data and Bayley II scores were similar between the groups. There was a trend towards less bronchitis (as reported by the parents) in the LISA intervention group (see Table 1).

**Table 1:** Follow-up results of the AMV-trial at 2-year corrected age

Parameter	Control *n* = 95	LISA Intervention *n* = 84	p
Age at follow-up [months]	27.1 ± 2.4	27.5 ± 3.4	0.37
Body length [cm]	85.7 ± 4.3	85.8 ± 4.5	0.89
Body weight [kg]	11.6 ± 1.6	11.5 ± 1.8	0.64
Bronchitis in the last 12 months [%]	49	34	0.06
Ability to walk [%]	95	91	0.39
Bayley MDI	98.5 ± 16.6	92.0 ± 24.0	0.07
Bayley PDI	89.2 ± 18.7	87.8 ± 22.9	0.75

Somatic data and Bayley scores are mean ± SD Missing data of total 179 infants that underwent follow-up:body length *n* = 4, body weight n = 5, questionnaire (bronchitis) *n* = 17 MDI (mental development index) *n* = 52, PDI (psychomotor development index) n = 84

## Discussion

This is the first follow-up from a randomized controlled trial on LISA. We observed no relevant differences in weight, length or head circumference between the LISA intervention and the control groups at 2-year corrected age. In addition, psychomotor development and mental scales were similar. The mean MDIs (98.5 ± 16.6 vs. 92.0 ± 24.0) were lower than what is expected for a population of term newborns but in the expectable range for infants at 26 to 28 weeks of gestation (9). Ability to walk and need for hearing and/or visual aids (data not shown in detail) were also similar between the groups. In parent’s reports, there was a trend (*p* = 0.06) in the LISA group towards less often episodes of bronchitis, which may be a surrogate for improved lung function following LISA.

To date this is the largest study with longer-term data following LISA in the neonatal period. Follow-up data on infants after LISA are sparse. In 2010 Porath et al. from Cologne (10) compared 31 infants ≤ 27 weeks of gestation following LISA to a historical control cohort of 21 infants with standard therapy. At school age (median age at follow-up 6 5/12 years), the rate of infants without impairments was similar (42 vs. 38%). Another observational study from Teig and colleagues in Bochum, Germany (11), compared 53 infants ≤ 28 weeks after the introduction of LISA in their unit to a control group of 44 infants prior to LISA therapy. Fifty-two percent of discharged infants were assessed for neurodevelopmental outcome at corrected age of 3 years. Mental development index (MDI, 89 vs. 98, *p* = 0.16) and psychomotor development index (PDI: 83 vs. 91, *p* = 0.03) at 3 years improved between the 2 periods. However, the authors concluded that the observed trends for better pulmonary and neurocognitive outcomes in a retrospective study from different time periods should be interpreted with caution until results from randomized trials on the LISA procedure are available. A larger unpublished outcome study from Vienna also based on a cohort with historical controls (12) points into the same direction. Data reported in abstract form from the 5-year follow-up of LISA infants in the GNN cohort suggest better lung function (FEV_1_) and better neuro-outcome/intellectual properties (WPPSI score) in infants that received surfactant via LISA compared to infants that received surfactant via the standard route (13). Again, all these studies and 2 recently published studies from Spain (14, 15) using historical controls were non-randomized, so that selection bias is likely to account for part of the positive results that were observed in favour of LISA.

However, on closer analysis of our data, it turned out that the adherence rate to the new regulations was lower than expected (Fig. 1 and Table 1). Formally, more than 80% of the infants were available for the follow-up, but not all infants were tested with Bayley II both in terms of mental development index (MDI) and especially the psychomotor development index (PDI). In addition, we found that the variance in Bayley scores was high (large SD) within but also between the different participating centres (Table 1). There seems to be a need for better standardization of the follow-up, e.g. in future studies, infants that cannot perform the tests should not be counted as missing values but with a result of < 2 SD.

In consequence, for the ongoing school age follow-up of the NINSAPP study (16), a randomized controlled study following the AMV study with a similar design but including infants from 23 to 26 weeks of gestational age, one team of investigators now travels to the different study sites. Hence the investigators are blind to the study group allocation, and the equipment (somatic measures, lung function (spirometry), exercise tests (3 min running test), blood pressure, hearing test, visual acuity, neurological investigations and psychomotor function tests) used is identical for all infants. In addition, a group of healthy term newborns is investigated as a mature control group, and for the school age follow-up interviews, the items picked are identical to a large German study health on infant health (https://www.kiggs-studie.de) which will allow comparability to a normal term newborn/paediatric/youth cohort.

In conclusion, the first follow-up study of LISA-treated premature infants from a randomized controlled study underlines the safety of this novel less invasive approach. Future studies should include long-term follow-up preferably at least until school age.

## Abbreviations

AMV: Avoid mechanical ventilation
BPD: Bronchopulmonary dysplasia
CPAP: Continuous positive airway pressure
CRF: Case record form
FEV_1_: Forced expiratory volume in 1 second
FiO_2_: Fraction of inspired oxygen
GBA: Gemeinsamer Bundesausschuss
GNN: German Neonatal Network
INSURE: Intubate surfactant extubate
IVH: Intraventricular haemorrhage
LISA: Less invasive surfactant administration
MDI: Mental development index
NINSAPP: Non-intubated surfactant application
PDI: Psychomotor development index
VLBW: Very low birth weight infants
WPPSI: Wechsler Preschool and Primary Scale of Intelligence
